# Clinical Efficacy of Treatment of Endodontic-Periodontal Lesions: A Systematic Scoping Review of Experimental Studies

**DOI:** 10.3390/ijerph192013649

**Published:** 2022-10-21

**Authors:** Carlos M. Ardila, Annie Marcela Vivares-Builes

**Affiliations:** 1 Basic Studies Department, School of Dentistry, Universidad de Antioquia UdeA, Medellín 050010, Colombia; 2 School of Dentistry, Institución Universitaria Visión de Las Américas, Medellín 050031, Colombia

**Keywords:** endodontic-periodontic lesion, combined periodontal-pulpal lesions, treatment efficacy

## Abstract

Background: In this review, we evaluated the clinical efficacy of interventions used for the treatment of endo-perio lesions (EPL). Methods: A systematic scoping review of clinical trials was developed. Results: Seven clinical trials were studied. In at least one study group of six of the seven evaluated trials, endodontic and periodontal treatments were performed simultaneously. All trials showed improvement in probing depth in the studied groups; nevertheless, the experimental groups of five studies demonstrated a statistically significant reduction in this parameter. An increase in clinical attachment level was also reported (*p* < 0.05). Most trials reported an increase in the filling of the bone defect following therapy (*p* < 0.05). Mechanical periodontal therapy implemented simultaneously with endodontic therapy demonstrated efficacy in the treatment of concurrent EPL without communication. Endodontic treatment and immediate periodontal surgery did not affect the result of the management of combined EPL with apical communication. The use of diode laser, the management of platelet-rich fibrin (PRF) and titanium-prepared PRF, and the implementation of bone grafts plus endodontic treatment with mineral-trioxide or gutta-percha seems to be an adequate strategy in patients with EPL. Conclusions: The treatment of EPL using simultaneous endodontic and periodontal therapies seems to be an acceptable treatment alternative.

## 1. Introduction

The periodontium and the dental pulp communicate through anatomical (exposed dentin, accessory canals, and the apical foramen) or pathological (root fractures) forms. These communications allow for the appearance of lesions that concurrently affect the periodontal and pulpal tissues, called endodontic-periodontal (endo-perio) lesions (EPL) [1]. These lesions can compromise patients with or without periodontitis according to a recent classification, which also indicates that the primary origin (endodontic or periodontal) is not crucial for treatment [2].

Therapeutic challenge of EPL includes the eradication of bacteria located in periodontal tissues and root canals. Hence, multiple therapeutic options that include endodontic and periodontal management have been proposed. However, it has been indicated that protocols for treating concurrent EPL demand more evidence-based investigations, as they are established mainly on the basis of retrospective studies [3]. Unfortunately, clinical evidence of the efficacy of these interventions through the evaluation of clinical trials has not resulted in a consensus. Therefore, assessing the best-available scientific evidence through clinical trials will allow clinicians to make better decisions to implement these outcomes in their practice. In this context, it is relevant to carry out a systematic scoping review of clinical trials to evaluate the efficacy of EPL treatments.

The objective of this systematic scoping review is to evaluate the clinical efficacy of interventions used for the treatment of EPL in terms of probing depth (PD), clinical attachment level (CAL), and bone defect fill.

## 2. Materials and Methods

### 2.1. Information Sources and Search Strategy

A systematic scoping review of clinical trials was developed considering the PRISMA (Preferred Reporting Items for Systematic Reviews and Meta-analyses) extension for scoping reviews [4]. PubMed/MEDLINE, SCOPUS, SCIELO, and LILACS databases were reviewed, in addition to the gray literature. Keywords and MeSH terms were used to investigate clinical intervention studies in all languages published until August 2022, including the terminologies endo-perio lesions, endodontic-periodontal lesions, combined periodontal-pulpal lesions, endo-perio treatment, endodontic-periodontal treatment, treatment efficacy, and randomized clinical trials (RCTs) and comparative clinical studies. Then, an exploratory process was implemented to review databases utilizing Boolean operators (AND, OR): “endo-perio treatment” OR “endodontic-periodontal treatment” AND “clinical trials” AND “clinical intervention studies” OR “prospective comparative clinical studies” OR “RCTs”.

### 2.2. Eligibility Criteria

Inclusion criteria: Only patients with non-vital tooth and EPL with a follow-up after therapy of at least 6 months were included. Moreover, only prospective trials were incorporated.

Exclusion criteria: Investigations that included lactating and pregnant women, periodontally treated patients (6 months before the study), previous root fillings, fractured/perforated roots, unrestorable tooth, inflammatory root resorption, patients with systemic conditions consuming antimicrobial or immunosuppressive medicines two months before the research, and smokers were not included. Retrospective investigations, case reports, case series, in vitro and animal studies, and duplicate publications were also not considered.

### 2.3. Research Questions

The aim of this systematic scoping review is to respond to the following question. In patients presenting combined EPL, what is the efficacy of (a) different endodontic treatments; (b) different periodontal treatments; and (c) the timing between them in terms of PD reduction, CAL gain, pocket closure, and bone defect fill?

### 2.4. Data Selection

Selected keywords utilized by both investigators occasioned the choice of the articles based on the reading of abstracts and full texts. Subsequently, the two researchers selected the trials according to the previously established inclusion criteria. Later, all abstracts and full texts were downloaded and separately assessed. The eligibility conditions were implemented to classify the papers to be included in this systematic scoping review. In the case of discrepancy among the investigators, study acceptability was determined by agreement. The Kappa test was implemented to formulate a score of agreement between researchers (>92).

### 2.5. Data Extraction

All full texts that met the inclusion criteria were read individually by both authors and assessed to prepare this systematic scoping review. A table was generated incorporating the most important information from the selected studies (autonomously by each of the investigators), and the results were contrasted. The table includes authors’ names; date of publication; number of patients; main characteristics of the methodology; diagnoses; and clinical efficacy in terms of PD, CAL, pocket closure, and bone defect fill. 

### 2.6. Outcome Measures

The primary outcome was change in PD. The secondary outcomes were changes in CAL, pocket closure, and bone defect fill. 

### 2.7. Risk of Bias

Both authors separately estimated the methodological quality of the included studies using a previously validated instrument that contains 16 criteria [5]. The authors were required to award each study a score on a scale from 0 to 3 for each of the criteria. Where authors failed to present the level of detail needed to draw a conclusion for a point, a score of 0 was conferred for that item. When there a level of certainty was presented for the evaluated item, a value of 3 was assigned. When results were unclear, a value of 2 was assumed. The sum of these conditions offers a total outcome for the body of evidence, quantified as a percentage of the highest possible score (100%).

## 3. Results

**Figure 1:**
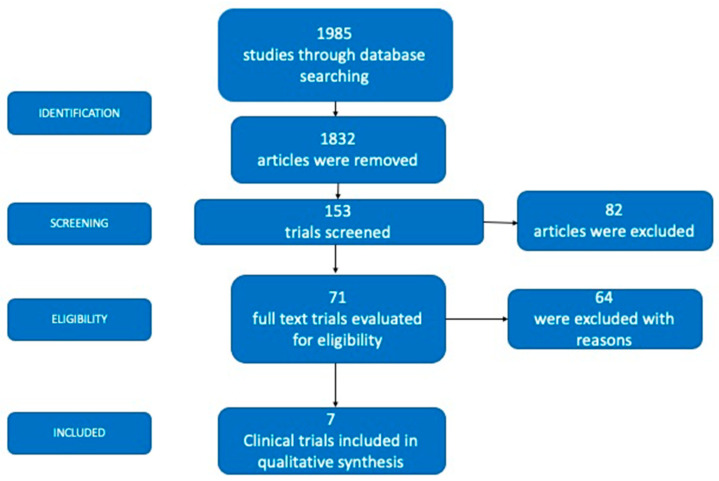
Flow chart of the study selection method.

The initial electronic search yielded 1985 studies, of which 1832 were excluded because they were not experimental studies. After reviewing the titles and abstracts, an additional 82 investigations were excluded. Reading the full text resulted in the exclusion of another 64 studies. Ultimately, seven clinical trials were included in this systematic scoping review (Figure 1). 

The characteristics of the included studies are presented in Table 1. These studies were published between 2012 [6] and 2022 [7]. The investigations assessed 739 teeth in 730 patients, with a minimum sample of 12 patients [7] and a maximum of 327 patients [8]. 

Table 1 depicts the different treatment modalities for EPL. Multiple therapies were performed, including endodontic and periodontal approaches with non-surgical and surgical methods, in addition to the use of different materials for endodontic and periodontal treatment. Researchers compared the treatment efficacy of EPL using endodontic treatment and scaling and root planing (SRP) performed simultaneously versus SRP performed 3 months after endodontic treatment [3], a standard treatment protocol and a standard diode laser-assisted treatment [6,7], gutta-percha (GP) and mineral trioxide (MTA) as an obturation material alone and with the addition of bone grafting [8], root canal treatment plus periodontal treatment versus endodontic treatment plus supragingival scaling [9], endodontic treatment plus the platelet-rich fibrin (PRF) versus endodontic treatment plus titanium-prepared platelet-rich fibrin (T-PRF) [10], and open flap debridement (OFD) performed 21 after initiation of endodontic treatment plus SRP versus OFD performed 3 months after initiation of endodontic treatment and SRP [10] (Table 1). It is important to note that endodontic and periodontal treatments were implemented simultaneously in most trials [6,7,8,9,10]. Razi et al. [1] performed the endodontic treatment first. Similarly, Gupta et al. trial completed endodontic treatment first. SRP was implemented 3 months after endodontic treatment. Endodontic and periodontal treatments were performed simultaneously in the control group [3]. 

Two studies were non-randomized clinical trials [1,9], and five were RCTs [3,6,7,8,10]. All studies had a follow-up period of between 6 and 24 months.

Because a new classification of EPL has been recently introduced [2], the diagnoses presented in the included articles are reflected as follows: endo-periodontal lesion in periodontitis patients in three trials [7,9,10] and endo-periodontal lesion in non-periodontitis patients in the remaining four experiments [1,3,6,8].

The clinical efficacy of the treatments performed on EPL was evaluated using the outcome variables PD, CAL, and bone defect fill. Pocket closure was not reported in any of the reviewed studies.

In general, all the trials showed improvement in PD in the studied groups; nevertheless, experimental groups of five studies [3,6,7,8,9] demonstrated a statistically significant reduction in PD (*p* < 0.05) (Table 1). 

Only four studies reported CAL results [1,3,6,10], showing gains in CAL in the studied groups studied without statistically significant differences between them, except for the study by Li et al. [6], which described greater improvement in the experimental group (Table 1). 

Regarding bone defect fill, researchers reported a reduction in bone loss following therapy [3,6,7,8,9]. Two RCTs reported a greater reduction (*p* < 0.05) in the experimental groups [7,8]. AlJasser et al. [9] described that bone graft groups presented the highest defect fill level (100% and 97%). Two other RCTs found an improvement in the periapical index in the studied groups (*p* < 0.05) [3,6]. Yan et al. [8] described that the group managed with endodontic treatment plus supragingival scaling presented a significant reduction in bone resorption after two years of treatment (*p* < 0.05), an aspect that did not occur in the comparison group (improvement was observed in this group, but it was not statistically significant).

Three studies reported tooth mobility outcomes; two of them found no statistically significant differences between the evaluated groups [7,11], whereas one investigation reported a greater reduction in mobility in the experimental group (*p* < 0.05) [9].

All trials explored in this systematic review completely met at least 75% of the described quality standards [5]; consequently, they were cataloged as of good quality (Table 2). However, these investigations present considerable heterogeneity, mainly reflected in the interventions considered in each study, which makes it difficult to carry out a comprehensive statistical analysis.

## 4. Discussion

To the best of our knowledge, this systematic scoping review is the first to evaluate the clinical efficacy of interventions for EPL through clinical trials. A systematic review published almost 10 years ago included retrospective studies, series, and case reports, considerably detracting from its level of scientific evidence [11]. Moreover, the management of combined EPL needs to be evaluated with more clinical trials [10]. 

Approximate 50% of tooth loss is produced by either endodontic infection, periodontitis, or the combination of the two in the form on EPL [12]. Consequently, these types of lesions require considerable attention for their management considering the best available scientific evidence. 

The management of EPL is challenging for clinicians because periodontal and endodontic therapy must be finished to guarantee an effective clinical result. The treatment of EPL is complex because the clinical procedure itself is difficult, including the meticulousness of the sequence of procedures and the choice of the appropriate materials [8]. As noted in this review, initial endodontic treatment before periodontal therapy was only performed in two of the reviewed trials [1,3]. This allows for microbial control inside the root canal, preventing it from affecting the result of periodontal therapy [8,13]. However, Gupta et al. [3] observed that in the control group, the simultaneous performance of periodontal and endodontic therapies allowed for improvement of periodontal parameters earlier. Other trials in which periodontal and endodontic treatments were performed concurrently also reported improvement in periodontal parameters [6,7,8,9,10]. These results will be contrasted below.

The clinical trials reviewed here describe distinct possibilities for endodontic therapy. Likewise, depending on the bone defect formed, diverse alternatives are available for periodontal treatment (Table 1). These strategies include diode laser-assisted treatment, GP, MTA, SRP, supragingival scaling, bone grafting, PRF, T-PRF, and OFD. Treatment options and material innovation have changed significantly over the years, making it difficult to compare these investigations. 

The decrease in PD provides an alternative endpoint, which could represent a success rate from a periodontal point of view. In this review, a reduction in PD was observed in re-evaluated teeth during the follow-up periods in each study, both in the experimental groups and in the control groups. However, four trials presented a statistically significant reduction in the experimental groups (*p* < 0.05) [6,7,8,9] and one in the control group [3]. This difference was similar (1.8 mm on average) for four of the studies [6,7,8,9] and 1.1 mm for the remaining study in the first three months of follow-up [3]. On the other hand, in a systematic review, Schmidt et al. [11] reported that a PD decrease was accomplished in practically all re-evaluated teeth, although there may be residual deep PDs; nonetheless, the PD of the studied teeth diverged notably among the clinical investigations. Restoration of periodontal health after SRP may take several months, especially in deeper pockets. As observed in the trials included in this review, re-evaluation times of at least 6 months are recommended [11]. On the other hand, it has been recognized that in addition to PD reduction, it would be clinically relevant to assess which pockets achieved the endpoint of therapy [14]. Unfortunately, in the trials included in this review and as reported in the systematic review by Citterio et al. [14], pocket closure is rarely reported as an outcome. Reporting pocket closure as a result is essential and should be included in future investigations [15].

Herein, an improvement in CAL was also reported in four trials [1,3,6,10] but without a statistical significance among groups, except for the studies by Gupta et al. [3] and Li et al. [6]. Gupta et al. [3] showed an improvement in CAL at three months of follow-up in the group in which endodontic treatment and SRP were performed simultaneously, whereas Li et al. [6] presented an improvement in the experimental group. Unfortunately, CAL was poorly evaluated in studies of EPL, which makes it difficult to contrast these results. However, it has been reported that CAL is a parameter that adequately indicates periodontal health [15]. More clinical trials evaluating CAL in the treatment of EPL are required. 

Most of the studies reviewed here evaluated bone defect fill after treatment of EPL, reporting appreciable improvement in all studied groups [3,6,7,8,9]. Two RCTs described greater improvement in the experimental groups [7,8]. Dembowska et al. indicated that the use of a diode laser has a relevant effect in reducing bone loss [7]. Laser therapies have been proposed to decontaminate and prepare the root canal, eliminate pathogens located in the periodontal pockets, and avoid surgical therapy [16,17]. It has been suggested that its effectiveness is due to the fibers’ access to the furcation, deep pockets, and root cavities [18]. On the other hand, AlJasser et al. reported that bone grafting in addition to obturation with MTA provides greater efficacy in the management of EPL [9]. The outcomes of obturation with MTA have been described as favorable; MTA presented a better performance in challenging endodontically complicated teeth with large pathosis. Moreover, the considerable achievement rate with MTA and bone grafting may be due to the positive physical features of MTA concerning hard tissue deposition and the regenerative method with bone grafting, which accelerates cell differentiation/proliferation/induction and tissue development [19]. 

One strength of this systematic scoping review is the longitudinal description of the involved trials, although considerable methodological dissimilarities were detected among them. Although only two RCTs reported dropouts [3,11], no study described tooth loss. Moreover, pocket closure was not reported. The exclusion of hypermobile and multi-rooted teeth appears to be of prognostic significance and could have repercussions for the therapy result. Consequently, therapy failures can be underrepresented. A similar situation was observed in a previous review [12]. Another issue of importance is that the trials studied in this review mostly had a follow-up time of between 6 and 12 months. The time required for full periapical and periodontal healing could be longer. For this reason, longer follow-up times are desirable.

## 5. Conclusions

Considering the limitations of the current systematic scoping review, we found that in most of the reviewed studies EPL was treated simultaneously with endodontic and periodontal therapies. In general, although multiple treatment alternatives were presented in this review, improvement was reported in periodontal parameters, such as probing depth, clinical attachment level, and filling of bone defects.

## Figures and Tables

**Table 1:** Characteristics of the evaluated experiments.

Authors	Diagnoses	Participants/Number of Affected Teeth	Mean Age	Female/ Male	Endodontic Intervention	Periodontal Intervention	Main Outcomes	Follow-Up
Dembowska et al. [7]	Periodontitis stage III. Endo-perio lesion.	12/12	47 years	5/7	Experimental group: rotary and hand instruments were implemented using the crown-down method. An Epic X Biolase diode laser was used at 940 nm twice a month for three months. Calcium hydroxide paste was applied to the canals between visits. After three months, the canals were filled with GP cones by lateral condensation. Control Group: rotary and hand instruments were applied to the canals between visits without a diode laser. After three months, the canals were filled with GP cones by lateral condensation.	Experimental group: SRP using an ultrasonic scaler and hand curettes plus laser-inactive tip. Three repetitions were performed in each pocket, with intervals of 10 s, twice a month for three months. Control group: SRP using an ultrasonic scaler.	Differences were observed in the decrease in PD between the experimental group and the control group, favoring the experimental group (1.88 ± 0.4 mm versus 0.23 ± 0.09 mm; *p* < 0.05). Tooth mobility in the experimental group decreased from 1 to 0 (*p* > 0.05). There was a greater increase in bone level in the experimental group (52.5% versus 27%; *p* < 0.05).	6 months
AlJasser et al. [9]	An upper anterior non-vital single-rooted tooth with true combined endo-periodontal lesions	120/120	41 years	95/53	Apical-coronal techniques were prepared with hand K-files at the established working lengths. Sizes 3, 4, or 5 reamer obturation, injection of the thermo-plasticized GP was performed twice, separately in the control group and experimental group 2. The entire root canal system was filled with MTA for experimental groups 1 and 3.	Control group: SRP Experimental group 1: SRP Experimental group 2: SRP and grafting procedure to fill the bony defect. Experimental group 3: SRP plus grafting procedure to fill the bony defect.	At three months of follow-up, significant differences in mean PD values between groups were observed. PD values of patients in GP plus bone graft (experimental group 2) presented significantly higher PD values than the other three groups (*p* = 0.025). GP (control group) and MTA (experimental group 1) groups showed significantly higher PD values (4.8 ± 0.89 mm and 3.8 ± 0.75 mm, respectively) compared to groups that received bone grafting (3.1 ± 0.59 mm) (experimental groups 2 and 3). The bone graft groups (experimental groups 2 and 3) improved by 1.8 ± 0.4 mm, whereas the nongrafted groups improved by 0.7 ± 0.1 mm, on average. The MTA + bone graft group (experimental group 3) presented the highest defect fill level (100%), followed by the GP + bone graft group (97%) (experimental group 2).	12 months
Yan et al. [8]	Combined periodontal-pulpal lesions. Presence of endo-perio lesions without root damage.	327/360	48 years	171/156	Experimental group: ET Control group: ET	Experimental group: periodontal basic treatment for 2 weeks after ET. Six weeks later, if there were still more than 5 mm periodontal pockets and bleeding after detection, flap treatment was performed. Control group: supragingival scaling	The mean PD in the experimental group decreased by 1.8 ± 0.05 mm compared with the control group (*p* < 0.05). Two years after treatment, tooth mobility in the experimental group was significantly lower than that in the control group (*p* < 0.05). Alveolar bone absorption 2 years after operation was not significantly different from that before surgery (*p* > 0.05) in the experimental group. Alveolar bone absorption 2 years after treatment was significantly reduced compared with that before treatment (*p* < 0.05) in the control group.	24 months
Razi et al. [1]	Primary endo and secondary perio.	140/140	18–58 years	60/80	Experimental and control groups: ET was finalized for all the teeth studied prior to the periodontal treatment.	Control group: PRF in infrabony defect Experimental group: Titanium-prepared PRF in infrabony defect	Mean PD and CAL were improved after 3 and 6 months in both groups (*p* > 0.05). The mean change in PD after 6 months was 2.56 mm (42.59%) in the control group and 2.51 mm (43.90%) in the experimental group (*p* > 0.05). The mean change in CAL after 6 months was 2.52 mm (40.82%) in the control group and 2.41 mm (42.12%) in the experimental group (*p* > 0.05).	6 months
Tewari et al. [10]	Concurrent endo-perio lesion with apical radiolucency, along with communication through the periodontal pocket	40/40	42 years	8/32	Experimental and control groups: ET and intracanal medicament (calcium hydroxide) were placed for 7–10 days.	SRP with an ultrasonic scaler and hand instruments and ET were simultaneously performed. Control group (immediate periodontal surgery): OFD was performed 21 days after initiation of ET and SRP. Experimental group (delayed periodontal surgery): OFD was performed 3 months after initiation of ET and SRP.	Mean PD, CAL, and tooth mobility were improved after 3 and 6 months in both groups (*p* > 0.05). The mean change in PD after 6 months was 3.3 ± 0.54 mm in the control group and 3.4 ± 0.52 mm in the experimental group. The mean change in CAL after 6 months was 2.7 ± 0.12 mm in the control group and 2.69 ± 0.03 mm in the experimental group.	9 months
Gupta et al. [3]	Teeth with a clinical/radiographic diagnosis of a concurrent endo-perio lesion without communication	31/37	45 years	17/14	Experimental and control groups: step-back technique. Canals were obturated with GP with the lateral condensation technique.	Control group: SRP with an ultrasonic scaler and hand instruments. ET and SRP were performed simultaneously. Experimental group: SRP was performed 3 months after completing ET.	Both groups presented a significant improvement in all clinical parameters evaluated after the completion of endodontic and periodontal treatment (*p* < 0.05). However, there was more improvement in periodontal parameters in the control group at the 3-month follow-up compared with the experimental group (PD 1.35 ± 0.72 mm versus 0.21 ± 0.27 mm; *p* < 0.05; CAL 1.36 ± 0.72 mm versus 0.14 ± 0.32 mm; *p* < 0.05). At 3 and 6 months after SRP (3- and 6-month follow-up in the control group, and 6- and 9-month follow- up in the experimental group), both groups presented a similar reduction in PD and gain in CAL (*p* > 0.05). Improvements in periodontal parameters that were reached in 6 months in the experimental group were achieved only in 3 months in the control group (*p* > 0.05). An improvement in the periapical index score was observed in 100% of cases in both groups (experimental group =1.39 mm versus control group = 1.37 mm; *p* > 0.05).	6 months
Li et al. [6]	Endo-perio combined lesions	30/30	44 years	18/12	Control and experimental groups: ET	ET and SRP were performed simultaneously. Control group: SRP Experimental group: SRP plus diode laser irradiation	Mean PD and CAL were improved after 6 months in both groups. The mean change in PD after 6 months was 0.4 ± 0.04 mm in the control group and 1.67 ± 0.19 mm in the experimental group (*p* < 0.05). The mean change in CAL after 6 months was 0.59 ± 0.06 mm in the control group and 0.9 ± 0.08 mm in the experimental group (*p* < 0,05). An improvement in the periapical index score was observed in both groups (control group = 0.27 mm versus experimental group = 0.73 mm; *p* > 0.05).	6 months

GP = gutta-percha; MTA = mineral trioxide aggregate; SRP = scaling and root planing; PD = probing depth; CAL = clinical attachment level; PRF = platelet-rich fibrin; OFD = open flap debridement; ET = endodontic treatment.

**Table 2:** Quality of the chosen studies [5].

Study	a	b	c	d	e	f	g	h	i	j	k	l	m	n	o	p	Score
Dembowska et al. [7]	3	3	3	0	0	3	3	0	3	3	3	3	3	3	0	3	75%
Aljasser et al. [9]	3	3	3	3	0	3	3	3	3	3	3	3	3	3	0	3	87.5%
Yan et al. [8]	3	3	3	3	0	3	0	0	3	3	3	3	3	3	0	3	75%
Razi et al. [1]	3	3	3	3	0	3	3	3	3	3	3	3	3	3	0	3	87.5%
Tewari et al. [10]	3	3	3	3	0	3	3	3	3	3	3	3	3	3	0	3	87.5%
Gupta et al. [3]	3	3	3	3	0	3	3	3	3	3	3	3	3	3	0	3	87.5%
Li et al. [6]	3	3	3	0	0	3	3	3	3	3	3	3	3	3	0	3	81.2%

a. Explicit theoretical framework. b. Statement of aims. c. Research setting. d. Sample size. e. Representative sample. f. Description of the procedure or data collection. g. The rationale for choice of data collection. h. Detailed recruitment data. i. Statistical assessment of reliability. j. Fit between stated research question and method. k. Fit between stated research question and content of data. l. Fit between stated research question and method of analysis. m. Analytical method selected. n. Reliability of analytical process. o. User involvement in the design. p. Strengths and limitations.

## Data Availability

The data obtained in this review were pooled from the included investigations.
